# Development of a Medium-Density Genotyping Platform to Accelerate Genetic Gain in Fresh Edible Maize

**DOI:** 10.3390/plants15091288

**Published:** 2026-04-22

**Authors:** Jingtao Qu, Diansi Yu, Wei Gu, Yingjie Zhao, Kai Li, Hui Wang, Pingdong Sun, Felix San Vicente, Xuecai Zhang, Ao Zhang, Hongjian Zheng, Yuan Guan

**Affiliations:** 1 CIMMYT-China Specialty Maize Research Center, Crop Breeding and Cultivation Research Institute, Shanghai Academy of Agricultural Sciences, Shanghai 201403, China; qujingtao@saas.sh.cn (J.Q.); yudiansi@saas.sh.cn (D.Y.); guwei@saas.sh.cn (W.G.); zhaoyingjie@saas.sh.cn (Y.Z.); likai@saas.sh.cn (K.L.); wanghui@saas.sh.cn (H.W.); sunpingdong@saas.sh.cn (P.S.); fsanvi60@saas.sh.cn (F.S.V.); 2 International Maize and Wheat Improvement Center (CIMMYT), El Batan, Texcoco 56237, Mexico; xc.zhang@cgiar.org; 3 Shenyang City Key Laboratory of Maize Genomic Selection Breeding, College of Bioscience and Biotechnology, Shenyang Agricultural University, Shenyang 110866, China; zhangao7@syau.edu.cn

**Keywords:** fresh edible maize, GBTS, medium-density genotyping, genomic selection, genetic gain

## Abstract

Genotyping is a key step in molecular breeding. Due to its cost-effectiveness, accuracy, and flexibility, genotyping by target sequencing (GBTS) has become a preferred technology for medium-density genotyping. In this study, a new GBTS array for fresh edible maize was developed using resequencing data from 477 lines. The array contains 5759 SNPs evenly distributed across the maize genome, with average minor allele frequency (MAF) and polymorphism information content (PIC) values of 0.40 and 0.36, respectively. These SNPs are closely associated with 1566 functional genes. Cluster analysis of 198 maize lines based on the GBTS array was consistent with their pedigree relationships. Furthermore, 277 fresh waxy maize lines were genotyped and used for genomic selection analyses of hundred-kernel weight, kernel length, and kernel width. Comparative evaluation of different models indicated that Ridge Regression Best Linear Unbiased Prediction (rrBLUP) was the optimal model, with prediction accuracies of 0.33, 0.64, and 0.36, respectively. Additional analyses using different marker densities based on the rrBLUP model showed that prediction accuracy did not increase when the number of markers exceeded 2000, indicating that this array provides sufficient marker density for genetic analysis and genomic selection. Overall, this array provides a useful tool for genetic studies of fresh edible maize and facilitates the application of genomic selection in breeding programs.

## 1. Introduction

Maize (*Zea mays* L.) is one of the most important cereal crops worldwide and serves as a staple food, feed, and industrial raw material. Among its diverse types, fresh edible maize is widely consumed as a food and vegetable product and is favored by consumers worldwide. Fresh edible maize mainly includes sweet maize (*Zea mays* var. *rugosa*) and waxy maize (*Zea mays* var. *ceratina*) and sweet-waxy maize. Sweet maize appears to have originated in northern Mexico and was widely cultivated about 1000 years ago [1]. Sweet maize is a rich source of dietary fiber, folate, and provitamin A carotenoids, contributing to improved human nutrition and health. Sweet maize is characterized by mutations in key genes of the starch biosynthesis pathway (e.g., *shrunken2* and *su1*), which disrupt starch accumulation in the endosperm and consequently increase the levels of soluble sugars in the kernels [2,3,4]. Waxy maize appears to have originated in southwest China several hundred years ago and is distinguished from other maize by a recessive mutation in the *WAXY* gene that reduces synthesis of amylose in the kernel [5,6]. Compared with field maize, breeding of fresh edible maize focuses more on improving eating quality and nutritional quality traits. However, traditional phenotypic selection is inefficient for the rapid improvement of these complex traits. In addition, transgenic breeding faces limitations due to low public acceptance. Therefore, molecular breeding provides a more efficient and feasible strategy for the genetic improvement of fresh edible maize.

The development of molecular markers plays a crucial role in advancing molecular breeding of fresh edible maize. The types of genetic variations suitable for developing molecular markers are primarily divided into two categories. One category comprises variations based on polymorphism length, including Insertion and Deletion (Indel) and Simple Sequence Repeats (SSR). The other category consists of Single-Nucleotide Polymorphisms (SNP), which involve single nucleotide substitutions or insertions and deletions. SNP markers have been widely applied in genetic research due to their extensive distribution across the genome, strong genetic stability, and suitability for automated detection. KASPar, array-based, and the next-generation sequencing (NGS) technology are the major genotyping platforms. KASPar is a single-plex SNP genotyping platform and highly suitable for low-density SNP genotyping and has been widely used in maize for genetic studies [7,8]. The array-based genotyping technology fixes the SNP identity probe on the array and is helpful for the cross-project comparison [9]. The array-based genotyping technology suffers from limited flexibility, as the addition of new SNP markers requires redesigning. NGS technologies have been widely used to identify genome-wide and high-density genotypes. However, large-scale sequencing of breeding populations remains costly, making it impractical for routine applications in breeding programs.

Genotyping by target sequencing (GBTS) is a method for sequencing and identifying genotypes at specified target sites using multiplex PCR or probe capture [10]. GBTS has been widely applied in plant and animal genetic research due to its advantages of high throughput, high accuracy, lower cost, and greater flexibility [11]. GBTS was first reported in maize with development of four arrays containing 1 K, 5 K, 10 K, and 20 K SNP markers [10]. Subsequently, GBTS has been successfully applied to major crops such as rice, wheat, and soybean [12,13,14,15].

Genomic selection (GS), first proposed by Meuwissen, refers to the use of both genotypic and phenotypic data from a training population to estimate the genetic effects of genome-wide markers [16]. These estimated effects are then applied to the genotypic data of individuals with unknown phenotypes to predict their genomic estimated breeding values (GEBVs), which can be used for breeding selection. As one of the core technologies in the Breeding 4.0 era, GS has been successfully applied to major cereal crops such as maize, rice, and wheat [11,17,18]. GBTS is considered one of the most suitable genotyping technologies for GS applications.

A large number of GS models have been developed, which can be broadly classified into three categories: linear models, Bayesian methods, and machine learning approaches [19]. Linear models, such as genomic best linear unbiased prediction (GBLUP) [20] and ridge regression best linear unbiased prediction (rrBLUP) [21], are widely used statistical approaches. These models estimate phenotypic values of individuals by constructing a genomic relationship matrix (G) based on genome-wide markers, offering advantages of stable prediction accuracy and high computational efficiency. Bayesian methods, including BayesA, BayesB, BayesC, Bayesian least absolute shrinkage and selection operator (Bayesian LASSO), and Bayesian Ridge Regression (BRR), assume that marker effects follow specific prior distributions and estimate their effects accordingly [22,23]. These models are known for their flexible model design and relatively high prediction accuracy. However, both linear models and their corresponding Bayesian approaches are primarily based on the assumption of additive marker effects, which limits their ability to capture complex non-linear relationships between genotype and phenotype. In contrast, machine-learning-based GS models, such as reproducing kernel Hilbert space (RKHS) [24], support vector machines (SVM) [25], and kernel ridge regression (KRR) [26], can effectively model non-linear relationships between genotypes and phenotypes, thereby improving prediction performance in complex traits. Nevertheless, these approaches often suffer from limited interpretability, making it difficult to quantify the contribution of individual markers.

Unlike field maize, which is primarily bred for grain yield and disease resistance, fresh edible maize breeding mainly targets eating quality and nutritional quality, resulting in a distinct genetic background compared with field maize. Although numerous GBTS arrays have been developed for maize [10,27,28], they were all designed based on the genetic background of field maize, thereby limiting the progress of molecular breeding in fresh edible maize. In this study, we designed a medium-density GBTS array from 477 diverse germplasm resources currently used in a fresh edible maize breeding program and evaluated its application in genetic analysis and GS for fresh edible maize. This study will be useful for accelerating genetic gain in fresh edible maize.

## 2. Results

### 2.1. Characteristics of Resequencing Data and Variants

In this study, whole-genome resequencing was performed on 477 maize inbred lines, generating a total of 6797.91 Gb of raw reads. The raw read size of each sample ranged from 9.48 to 22.97 Gb, with an average of 14.25 Gb. After quality control, 6733.29 Gb of high-quality clean reads were retained, accounting for 99.05% of the raw data. The clean reads size of each sample ranged from 9.38 to 22.85 Gb, averaging 14.12 Gb. Of these, the size of 442 samples (92.66% of the total) was more than 11 Gb of clean reads. Based on the estimated maize reference genome size of 2.2 Gb, this data size corresponds to a sequencing depth of ≥5×. Notably, although low-depth sequencing may reduce the sensitivity for detecting heterozygous SNPs, the large sample size and stringent filtering criteria effectively mitigate this limitation, thereby ensuring the overall reliability of SNP discovery. Detailed information on the whole-genome resequencing data is provided in Table S1.

Following variant calling, a total of 101,471,606 raw SNPs were identified. These variants were broadly distributed across all ten maize chromosomes, providing a rich source of genetic variation for the development of a medium-density fresh edible maize genotyping chip. In addition, these high-density genotype data provide valuable resources for genetic studies, such as genome-wide association studies. After quality control analysis, a total of 1,266,886 high-quality SNPs was obtained.

### 2.2. Characteristics of the Fresh Edible Maize 5K SNP Array

**Figure 1:**
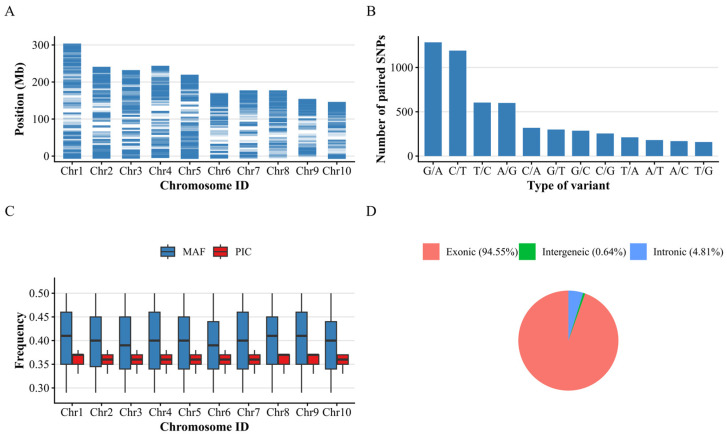
Characteristics of the fresh edible maize 5K SNP array. (**A**) The distribution of 5k markers on ten maize chromosomes. (**B**) Types of variation among the 5k SNPs in the array. (**C**) Boxplot of MAF and PIC across the 477 fresh edible maize population for 5K SNP array. (**D**) The genomic distribution of SNPs across functional regions.

**Figure 2:**
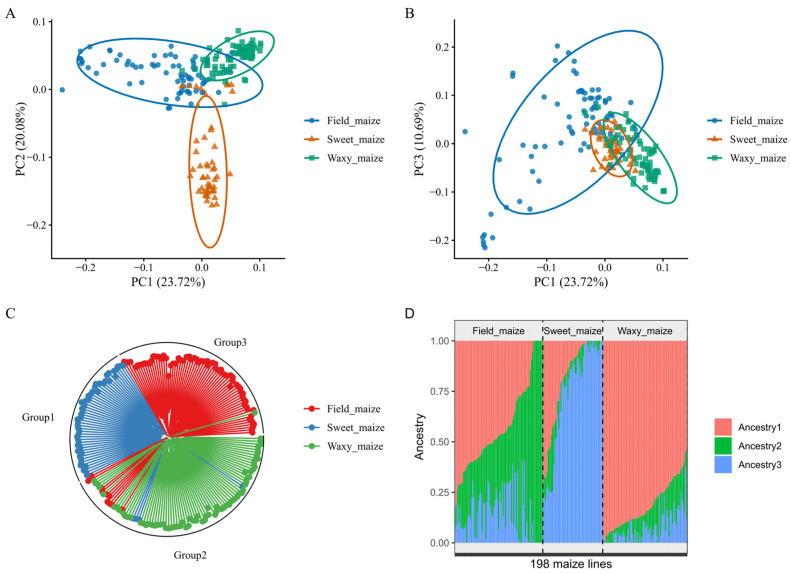
The principal component analysis, NJ tree, and population structure in 198 maize lines. (**A**) indicates PC1 versus PC2. (**B**) indicates PC1 versus PC3. (**C**) indicates the circular NJ tree in the population. (**D**) indicates the population structure in the population (K = 3).

Following probe design and evaluation, a total of 5759 SNPs were retained from all high-quality SNPs for the medium-density genotyping platform (Table S2). These SNPs were uniformly distributed across the ten chromosomes of the maize genome (Figure 1A). The number of SNPs on each chromosome ranged from 365 on chromosome 7 to 856 on chromosome 5, with a mean of 575.90 SNPs per chromosome (Figure 2, Table 1). Additionally, the average distance between adjacent SNPs on each chromosome ranged from 261.57 Kb on chromosome 5 to 499.68 Kb on chromosome 7, with a mean of 365.75 Kb.

The SNP mutation type analysis indicated that 66.22% of the SNPs were transition (A/G and T/C), while 19.29% were A/C and T/G, and the remaining 14.48% were A/T and G/C (Figure 1B). The number of SNPs with A/G and T/C allelic types was significantly higher than that of other mutation types. The distribution of SNP mutation types in this chip is consistent with the transition bias observed in existing low- and medium-density genotyping platforms in maize [29,30], further supporting the rationality of SNP marker selection in this chip.

The MAF of these SNPs across the 477 fresh edible maize lines ranged from 0.29 to 0.50, with a mean of 0.40, whereas the PIC ranged from 0.33 to 0.38, with a mean of 0.36 (Figure 1C). The missing rate of these SNPs across the 477 fresh edible maize lines ranged from 0 to 1.81%, with a mean of 0.12%, and 5712 SNPs (99.18%) had a missing rate < 1%. Additionally, functional annotation showed that most SNPs were located in exonic regions (Figure 1D). Of the 5759 SNPs, 5445 were located in exonic regions, 277 in intronic regions, and only 37 in intergenic regions. Notably, the relatively high proportion of exonic SNPs can be attributed to the prioritization of functionally relevant variants during marker selection, as these loci are more likely to be associated with phenotypic variation and thus are advantageous for downstream genomic prediction. These 5759 SNPs were associated with 1566 genes. Specifically, 5719 SNPs were located in the gene regions of 1332 genes, 291 SNPs were within 2 kb upstream of 96 genes, and 566 SNPs were within 2 kb downstream of 172 genes. The gene function of all 1566 genes is provided in Table S3.

These results highlighted the high quality of the 5759 selected SNP markers, which showed consistent genotyping performance, low levels of missing data, and appropriate minor allele frequencies, making them suitable for genetic analyses and the development of a medium-density fresh edible maize genotyping chip.

### 2.3. The 5K SNP Array for Genetic Analysis

To test the developed SNP array, 198 randomly selected maize lines including fresh edible sweet maize lines, fresh edible waxy maize lines, and field maize lines were used for genotype detection (Table S4). The call rate of individual samples ranged from 90.54% to 98.45%, with a mean of 96.64%. Additionally, 164 samples (83.33%) exhibited a call rate greater than 95% (Figure 2A). The array exhibited a high sample call rate, indicating that it is robust and reliable for genotyping diverse fresh edible maize germplasm resources. After filtering, a total of 4829 high-quality SNPs were retained for further genetic analyses.

The result of principal component analysis (PCA) in all of the 198 maize lines is shown in Figure 2A,B. The first three principal components explained 54.48% of the genetic variations, and the genetic variant explained by PC1, PC2, and PC3 was 23.72%, 20.08%, and 10.69%, respectively. A small number of sweet maize inbred lines and waxy maize inbred lines could not be clearly separated from grain maize, likely due to germplasm introgression between fresh edible maize and grain maize populations during the breeding process. Overall, a clear genetic divergence was observed among the sweet maize inbred lines, waxy maize inbred lines, and field maize inbred lines.

Further phylogenetic analyses based on the genetic distance matrix clearly separated the 198 maize lines into three groups (Figure 2C). Group 1 consisted entirely of sweet maize inbred lines, Group 2 was mainly composed of waxy maize inbred lines, and Group 3 was predominantly made up of field maize inbred lines. The clustering result was highly consistent with their pedigree classifications. Notably, four sweet maize inbred lines and ten field maize lines were assigned to Group 2, and one waxy maize line was assigned to Group 3. These results indicate that, within the selected validation population, the improvement of waxy maize primarily involved the use of grain and sweet maize germplasm, which is consistent with the actual breeding practices of our team.

The optimal K was selected using the “elbow method” based on the cross-validation (CV) error. The CV error decreased substantially from K = 1 to 3, after which the rate of decline diminished and the error fluctuated without further improvement for K ≥ 4 (Table S5). The statistical indication of K = 3 as the elbow point is consistent with prior biological knowledge, as the 198 maize inbred lines are derived from three known breeding populations. Consequently, K = 3 was determined as the optimal number of ancestral populations. In the sweet maize group, the proportion of Ancestry 3 was significantly higher than that of the other two ancestral components. In contrast, Ancestry 1 predominated in the waxy maize group, whereas Ancestry 2 was more abundant than the other components in the field maize group (Figure 2D). These results indicate that Ancestry 2 is the primary factor underlying the genetic differentiation between fresh edible maize and field maize.

Collectively, these results indicate that the 5K SNP array for fresh edible maize can be used for genetic analysis. Additionally, the array could also be applied to field maize.

### 2.4. The 5K SNP Array for GS

**Figure 3:**
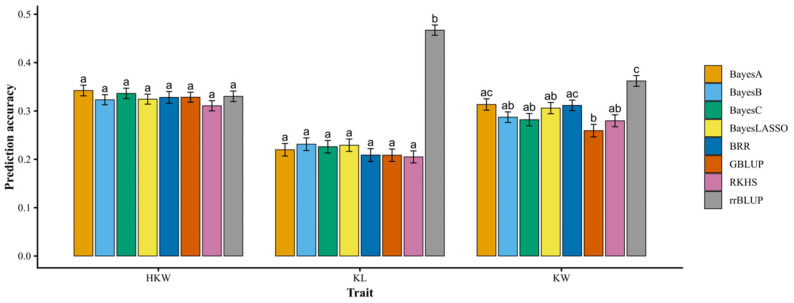
Prediction accuracy of different statistical models based on 5759 SNP markers. Error bars represent the standard deviation (SD) across 100 cross-validation replicates. Different letters (a, b, and c) indicate significant differences among models based on one-way ANOVA followed by Tukey’s HSD test (*p* < 0.05).

To evaluate the predictive performance of the 5K SNP array, eight different statistical models were used to predict three agronomic traits within the fresh edible waxy maize population comprising 277 inbred lines. Results indicated that prediction accuracy varied across traits (Figure 3). For 100-kernel weight, no significant differences were detected among the eight statistical models (*p* > 0.05). However, for kernel length and kernel width, significant differences in prediction accuracy were observed among models (*p* < 0.05). Notably, rrBLUP consistently achieved the highest prediction accuracy across traits, significantly outperforming the other seven models, highlighting its robustness and suitability for genomic prediction in this maize population.

**Figure 4:**
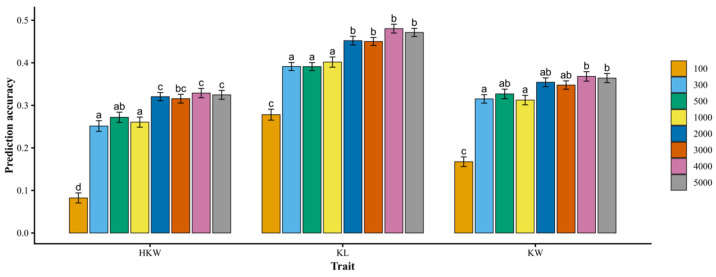
Prediction accuracy across different marker densities using rrBLUP model. Error bars represent the standard deviation (SD) across 100 cross-validation replicates. Different letters (a, b, c, and d) indicate significant differences among models based on one-way ANOVA followed by Tukey’s HSD test (*p* < 0.05).

Different marker densities were evaluated to assess their effects on the prediction accuracy of kernel-related traits in fresh edible waxy maize using the rrBLUP model. As the number of markers increased, prediction accuracy initially improved but reached a plateau when approximately 2000 markers were included. At this marker density, the average prediction accuracies for hundred-kernel weight, kernel length, and kernel width were 0.32, 0.45, and 0.35, respectively. Notably, no further significant increase in prediction accuracy was observed for any of the three traits when additional markers were included (Figure 4). These results indicate that a marker set of approximately 2000 SNPs is sufficient to achieve stable genomic prediction for kernel-related traits in fresh edible waxy maize using the present SNP array.

GS analysis in fresh edible maize indicated that the array is capable of delivering high-quality genotype data in a timely manner, providing a valuable tool for accelerating the improvement of fresh edible maize.

## 3. Discussion

In maize, a series of GBTS arrays have been developed and used in genetic studies, QTL mapping, and GS [10,27,28,29]. However, most GBTS arrays developed for maize are based on the genetic background of field maize. Since the breeding objectives of fresh edible maize differ from those of field maize, its genetic background may also differ considerably [31]. As one of the core strategies in the era of “Breeding 4.0,” GS requires large-scale genotyping for the development and selection of pure lines, making cost control a critical consideration [32,33,34]. In maize, most agronomic traits are complex quantitative traits controlled by numerous genes with small effects [35]. Therefore, low-density genotyping approaches (e.g., InDel, SSR) are often insufficient to achieve satisfactory prediction performance. In contrast, high-density genotyping platforms (e.g., whole-genome resequencing, GBS) provide comprehensive genomic information but are associated with high costs when applied to large breeding populations. Consequently, medium-density marker platforms represent an optimal compromise between cost efficiency and prediction performance, making them particularly suitable for large-scale GS applications in breeding programs. In the present study, a total of 5759 SNP markers were selected for development of a GBTS array for fresh edible maize. These SNPs are distributed across all ten maize chromosomes, which enhances the power of genetic analysis and genomic selection [34]. These markers exhibited relatively high levels of polymorphism, with mean MAF and PIC values of 0.40 and 0.36, respectively. High-polymorphism markers are essential for the effective implementation of GS, as they enhance the ability to capture genome-wide genetic variation and improve the accuracy of marker effect estimation [36]. The elevated MAF and PIC values observed here indicate strong allelic diversity and high discriminatory power within the fresh edible maize population. Importantly, all 5759 SNPs are biallelic loci, which facilitates their direct conversion into KASP markers [8]. This characteristic provides substantial flexibility for practical breeding applications, particularly in low-density genotyping scenarios such as DNA fingerprint construction, germplasm identification, and MAS. In terms of functional annotation, 5445 SNPs were located within exonic regions, involving 1556 annotated functional genes. SNPs situated in coding regions are more likely to be directly associated with functional variation or tightly linked to causal loci, thereby increasing their biological relevance [37,38,39,40]. Collectively, this SNP panel demonstrates strong genetic diversity, broad genome coverage, and functional significance, making it suitable for GS breeding, genetic diversity analysis, QTL mapping, and fingerprinting applications.

We applied the developed SNP array to genetic analyses of 198 maize inbred lines. The results demonstrated that the array performed robustly in PCA, phylogenetic tree construction, and population structure analysis, effectively capturing genetic relationships and population stratification. These findings suggest that the developed SNP array has the potential to be applied in the classification of heterotic groups in maize breeding, although further validation is required. Although the fresh edible waxy maize population used in this study was primarily composed of Chinese germplasm, the panel of 198 lines also included 76 field maize inbred lines originating from diverse global sources. This diversity suggests that the developed SNP array may have broader applicability beyond Chinese germplasm and could be useful across different ecological regions. However, further validation using more diverse populations is still required. We further applied the GBTS-based SNP array to genome-wide selection for kernel-related traits in 277 fresh edible waxy maize inbred lines and systematically compared eight statistical models. For HKW, no significant differences in prediction accuracy were detected among the models, suggesting that alternative assumptions regarding marker effect distributions did not substantially influence predictive performance for this trait. In contrast, for KL and KW, the rrBLUP model showed significantly higher prediction accuracy than the other seven models. These results indicate that KL and KW are typical highly polygenic traits primarily controlled by numerous loci with small additive effects, whereas the genetic architecture of HKW may be relatively more complex. The superior performance of rrBLUP, which assumes homogeneous marker variances and applies uniform shrinkage, suggests that additive genetic effects dominate the variation in kernel morphological traits in this population. Therefore, rrBLUP appears to be a robust and efficient model for genomic prediction in fresh waxy maize breeding programs.

To further evaluate the impact of marker density on predictive ability, different subsets of SNP markers were tested using the rrBLUP model. Prediction accuracy increased with marker number at lower densities but reached a plateau when approximately 2000 markers were included. Beyond this threshold, no significant improvement in predictive performance was observed. This plateau effect indicates that approximately 2000 well-distributed SNP markers are sufficient to capture most of the additive genetic variance in this population. This result is consistent with previous GS studies in maize [41]. Therefore, the medium-density array seems sufficient for the implementation of genomic selection in fresh waxy maize breeding, offering a cost-effective and scalable strategy for routine genomic selection programs.

The moderate prediction accuracies observed for hundred-kernel weight, kernel length, and kernel width are consistent with the quantitative and polygenic nature of these traits, which are typically controlled by many loci with small effects. Under such a genetic structure, the maximum achievable prediction accuracy using linear genomic prediction models may be inherently constrained. In addition, these traits are sensitive to environmental variation and genotype-by-environment interactions, and limited multi-environment phenotypic data may further reduce effective prediction accuracy. Therefore, the prediction accuracies reported in this study likely reflect both biological complexity and practical limitations of the available training data. Nevertheless, the developed GBTS5K platform still provides sufficient predictive ability for relative ranking and early-stage selection of breeding materials, demonstrating its practical value for genomic selection in fresh edible maize. Future improvements may be achieved by expanding the training population, incorporating multi-environment phenotypic data, and evaluating models that better capture non-additive genetic effects and genotype-by-environment interactions.

## 4. Materials and Methods

### 4.1. Plant Materials, Sampling, and Resequencing

477 diverse edible maize inbred lines were collected from the Maize Center of the Shanghai Academy of Agricultural Sciences for the development of a medium-density genotyping platform, including 170 sweet maize inbred lines, 286 waxy maize inbred lines, and 21 sweet-waxy maize inbred lines. The inbred lines were cultivated at the Zhuanghang Experimental Station of the Shanghai Academy of Agricultural Sciences. Genomic DNA was extracted from young leaf tissues of the inbred lines using the CTAB method. The integrity of the extracted DNA was assessed by 0.8% agarose gel electrophoresis, while the concentration and purity were measured using a NanoDrop spectrophotometer. Samples that passed the quality control were used for subsequent resequencing. The Illumina sequencing libraries were constructed with a size of 350 bp, and whole-genome resequencing was performed based on Illumina sequencing platform with paired 150 bp at NovoGene company (Beijing, China). Each sample was sequenced at a preset depth of more than 5×. The raw data from the sequencing platform were filtered using the FASTP (version: 0.23.2) software [42], retaining reads with a Phred quality score ≥ 20 (Q20). The clean data were mapped to the maize reference genome B73v4 (https://download.maizegdb.org/Zm-B73-REFERENCE-GRAMENE-4.0/Zm-B73-REFERENCE-GRAMENE-4.0.fa.gz, accessed on 1 May 2025) using bwa (version: 0.7.17) software with default parameters [43,44]. The variant calling was conducted using Freebayes (version: 0.9.21) software with default parameters [45].

### 4.2. Selection of SNP for Development of the Chip Array

The raw SNPs derived from the resequencing data were initially filtered using bcftools (version: 1.6) according to the following criteria: sequencing depth of between 2 and 50, genotyping quality ≥ 20, sample missing rate ≤ 0.05, biallelic loci only, and MAF ≥ 0.2. To account for LD and reduce SNP redundancy, SNPs were further pruned using PLINK (version: 1.9) with the parameter of “--indep-pairwise 50 5 0.2”, retaining a set of approximately independent SNPs for downstream analyses. High-quality SNPs located within genes and their flanking 2 kb regions were selected for GBTS probe design. The probes were designed using GenoBaits Probe Designer with a length of 110 nt and a GC content ranging from 30% to 70% [10]. The probe sequences were aligned to the reference genome B73v4 using BLAST (version: 2.12.0) to remove non-specific sequences. A two-cross-covered-probe strategy was employed for each SNP site. A random set of 6000 probe sequences was outsourced to MOLBREEDING Biotechnology Co., Ltd. (Shijiazhuang, Hebei, China) for probe synthesis, sample–probe hybridization, and Illumina sequencing.

MAF and missing rate were calculated using VCFtools (version: 0.1.16). PIC was calculated using the following formula:

$$PIC=1-\left(p^{2}+q^{2}\right)-2p^{2}q^{2}$$

where *p* and *q* are the population frequencies of the allele1 and the allele2.

### 4.3. Genotyping of 198 Maize Inbred Lines

To evaluate the developed GBTS5K chip, a panel of 198 maize inbred lines was genotyped. This validation population included 50 sweet maize inbred lines, 72 waxy maize inbred lines, and 76 field maize inbred lines. All 198 lines were obtained from the Maize Center of the Shanghai Academy of Agricultural Sciences. The plants were grown in a greenhouse until the five-leaf stage, and leaf tissues were sampled and flash-frozen in liquid nitrogen, and DNA was extracted via the CTAB method. The extracted DNA samples were genotyped using the GBTS5K chip following the procedure described in previous studies [46]. In this step, genomic DNA samples were randomly fragmented into 300–350 bp fragments, followed by hybridization capture using probes from the GBTS5K chip. The captured fragments were then used for library construction and sequenced on an Illumina platform with paired-end 150 bp. Raw sequencing data were subjected to quality control using fastp, followed by alignment to the reference genome using Bwa, and SNP calling was performed using GATK4 Best Practices pipeline with the specified genomic region. The genotype of 198 lines was filtered with quality of genotype ≥ 20, sample missing rate ≤ 0.05 and MAF ≥ 0.05. The high-quality genotype data was used to perform the principal component analysis (PCA) using PLINK (version: 1.9) with the parameter “--pca 10” [47]. The variance explained by each principal component was estimated by dividing its eigenvalue by the sum of the eigenvalues of all principal components. The pairwise genetic distances among the 198 maize inbred lines were calculated using TASSEL (version: 5.2.90), and a phylogenetic tree was subsequently constructed based on the genetic distance matrix using the neighbor-joining (NJ) method [48]. Population structure analyses of 198 maize lines was performed using Admixture (version: 1.3.0). The optimal K was determined using the “elbow method,” defined as the point where the decrease in cross-validation (CV) error markedly slowed. The results of PCA and population structure were visualized using the ggplot2 package in R (version: 4.5.2), while the phylogenetic tree was visualized with the ggtree package.

### 4.4. Genomic Prediction

Phenotypic data for 100-kernel weight, kernel length, and kernel width from 277 fresh edible waxy maize inbred lines, obtained from our previous study, were used for GS [49]. The population were genotyped using the array developed in this study. GS was performed with eight statistical models, including rrBLUP, GBLUP, BayesA, BayesB, BayesC, Bayesian LASSO, BRR, and RKH. These statistical models were performed using the two R packages, rrBLUP (https://CRAN.R-project.org/package=rrBLUP, accessed on 1 May 2025) with the default parameters and BGLR (https://CRAN.R-project.org/package=BGLR, accessed on 1 May 2025) with the parameters of “iterations = 12,000, burn-in = 3000”. The prediction accuracy was assessed using the five-fold cross-validation method with 100 replicates. The dataset was randomly divided into five subsets, with four subsets used as the training population and the remaining subset as the validation population in each iteration. This process was repeated 100 times to ensure robust and stable estimates of prediction accuracy. The Pearson correlation coefficient between the observed phenotypic values and the genomic estimated breeding values (GEBVs) in each cross-validation replicate served as a measure of prediction accuracy. Random subsets consisting of 100, 300, 500, 1000, 2000, 3000, 4000, and 5000 markers were sampled from the array to perform GS, aiming to assess whether the marker density is adequate for GS in fresh edible maize.

## Figures and Tables

**Table 1:** Distribution of 5759 SNPs across ten maize chromosomes.

Chromosome	Length (Mb)	Number of SNPs	Density of SNP (SNPs/Mb)	Average Distance (Kb)
Chr1	307.04	754.00	2.46	407.22
Chr2	244.44	735.00	3.01	332.57
Chr3	235.67	625.00	2.65	377.07
Chr4	246.99	576.00	2.33	428.81
Chr5	223.90	856.00	3.82	261.57
Chr6	174.03	524.00	3.01	332.12
Chr7	182.38	365.00	2.00	499.68
Chr8	181.12	380.00	2.10	476.64
Chr9	159.77	454.00	2.84	351.92
Chr10	150.98	490.00	3.25	308.13
Mean	210.63	575.90	2.73	365.75

## Data Availability

The original contributions presented in this study are included in the article. Further inquiries can be directed to the corresponding authors.

## Supplementary Materials

The following supporting information can be downloaded at: https://www.mdpi.com/article/10.3390/plants15091288/s1, Table S1: The resequencing information of 477 fresh edible maize germplasm; Table S2: The information of 5759 SNPs for the GBTS array; Table S3: The information of gene related to the 5759 SNP markers; Table S4: The genotyping data of 198 maize lines; Table S5: Cross-validation (CV) errors for K = 1 to 10 in ADMIXTURE analysis.
